# Association between Insufficient Sleep and Dental Caries among Preschoolers in Japan: A Cross-Sectional Multicentre Study

**DOI:** 10.3390/ejihpe12010001

**Published:** 2021-12-30

**Authors:** Masato Ogawa, Hiroto Ogi, Daisuke Nakamura, Teruo Nakamura, Kazuhiro P. Izawa

**Affiliations:** 1 Department of Public Health, Graduate School of Health Sciences, Kobe University, Kobe 654-0142, Japan; mogawa@med.kobe-u.ac.jp (M.O.); ogihiroto6062@gmail.com (H.O.); 2 Cardiovascular Stroke Renal Project (CRP), Kobe 654-0142, Japan; nakadai525@gmail.com (D.N.); nakamura_00_00@yahoo.co.jp (T.N.); 3 Fudousan Technologies Corporation, Sanda 669-1324, Japan; 4 Educational Corporation Tsukushi Gakuen, Chitose 066-0035, Japan

**Keywords:** sleep disorder, dental caries, preschoolers, children, parents

## Abstract

Recently, sleeping status has attracted attention for its relationship with oral health. In the present study, we have investigated the association between early childhood caries and sleeping status. A multicentre observational cross-sectional study was conducted among 332 preschoolers (aged 3–6 years) and their parents in Chitose, Japan. Dental caries and sleeping status were assessed in the children and the sleep quality and health literacy of the parents were also assessed. Univariate and multivariate regression analyses were used in order to investigate the effect of the sleeping status of the parents and their children on dental caries. Among the children, the prevalence of dental caries experience was 12.7%. The children without caries slept significantly longer and their parents had a better Pittsburgh Sleep Quality Index (PSQI) score than those with caries experience. The sleeping status and the numbers of caries in the children were significantly correlated. Health literacy was better in those without caries experience. Parents’ PSQI was significantly positively correlated with the numbers of caries in the children (r = 0.19, *p* = 0.0004). The children’s sleep durations, screen time, and parental smoking status were independently associated with early childhood caries. Poor sleeping status of children and their parents is related to dental caries among preschoolers.

## 1. Introduction

Oral health is important for a child’s well-being and development; dental caries, in particular, are a serious global public health issue. A previous study that was carried out by Ayhan et al. [1] demonstrated that children who had caries in their deciduous teeth were significantly shorter or weighed less than 80% of their ideal weight. Furthermore, Vinckier et al. [2] have stated that caries progression causes severe pulpitis and apical periodontitis in children due to delayed detection and treatment. Of note, the existing literature suggests that caries experience in the deciduous teeth is associated with a risk of subsequent caries or predisposes children to malocclusion in the permanent teeth [3,4,5]. Therefore, preventing early childhood caries is important during childhood as well as adulthood.

Risk factors for the development of dental caries in children are well-documented. A recent systematic review by Kirthiga et al. [6] revealed many risk factors for children’s dental caries, such as low education of the caregiver, presence of a single mother, less parental dedication to helping the child brush their teeth, presence of *Streptococcus mutans*, more limited parental education, frequent consumption of sweetened foods, and poor oral hygiene. Recently, Archer et al. [7] have reported that the relationship between caries and the impairment of the circadian rhythms due to irregular lifestyles has also attracted attention. Irregular lifestyles and sleep irregularities may decrease immune function and saliva flow, which may be risk factors for dental caries [8]. In a large cohort study in Japan, late bedtimes and short sleep durations were both consistently associated with an increased risk of caries in the deciduous teeth [9].

Parents play an important role in controlling the circadian rhythms and lifestyles of their children and there is a strong association between the oral hygiene behaviour of parents and the oral health-related habits of their children [10]. Nevertheless, the relationship between dental caries in children and parental sleep status is not clear. Thus, we hypothesised that sleep conditions of both parents and their children are related to early childhood caries. The present study aims to determine the relationship between early childhood caries and the sleep statuses of both parents and children.

## 2. Materials and Methods

### 2.1. Participants

This was a multicentre, cross-sectional study of 3 to 6 year-old preschool children in five kindergartens, nursery schools, and early childhood education and care centres in Chitose, Japan, as well as their parents. We included only the oldest child within families with two or more children in the same facility. To invite them to participate, we asked the school staff to distribute materials that included a description of the study’s intention, the consent for participation, the ability to withdraw consent to participate, and the questionnaire to parents. Participation was completely anonymous and voluntary and the final dataset was fixed after consultation with the heads of the facilities in October 2020. Exclusion criteria included children whose height or weight data were not measured in February 2020 and those missing their dental screening data. This study received prior approval from our institutional ethics committee. Informed consent was obtained from each parent via pamphlets and it was explained to them that they could withdraw their participation at any time. This study was conducted in accordance with the Strengthening the Reporting of Observational Studies in Epidemiology (STROBE) guidelines (Table S2).

### 2.2. Instruments

The data were self-reported by the parents for themselves and their children. The questionnaire included the following items for the children: age, sex, birthweight, number of siblings, breakfast (eaten every day or not eaten every day), extracurricular activities (yes or no), gaming and television hours, and total screen time (including television viewing and gaming on smartphones or computers) [11]. The body mass index (BMI) was calculated as the individual’s weight in kilograms divided by their height in metres squared. The questionnaire included the following items for the parents: age, sex, BMI, smoking and drinking habits, marital status (married or not), years of education, household income (≤6 million yen or >6 million yen), and comprehensive health literacy (HL). HL was assessed with the 14-item HL scale questionnaire [12] comprising 14 questions on a 5-point scoring scale. The scores range from 14 to 70, with higher scores indicating better HL.

The average hours of media use (television viewing, DVD viewing, and playing games on smartphones, computers, or consoles) per day on school days were categorised on a 6-point scale as follows: 1, no use; 2, less than 1 h; 3, 1–2 h; 4, 2–3 h; 5, 3–4 h; and 6, greater than 4 h.

### 2.3. Procedure

The oral health examination was conducted by only one dentist using a basic screening clinical examination procedure. The dental examination records included the number of decayed teeth and the number of restored teeth by fillings using the decayed–filled teeth (DFT) index for primary teeth [13]. The DFT index enables conclusions to be drawn regarding the history of caries in an individual.

The sleeping status of each child was assessed as sleep duration according to a questionnaire filled by their parents. Sleep problems in parents were assessed using the Pittsburgh Sleep Quality Index (PSQI) [14]. The PSQI is a widely used multidimensional self-report measurement questionnaire. The reliability and validity of the PSQI in Japanese have been demonstrated to detect sleep quality and disorders in adults and younger individuals [15,16]. The PSQI contains 18 questions assessing subjective sleep quality, sleep latency, sleep duration, habitual sleep efficiency, sleep disturbance, use of sleep medications, and daytime dysfunction. The total score of the PSQI is from 0 to 21. The higher the mean score, the poorer the sleep quality; the cut-off score for poor sleep quality was 5 points [14]. We used the PSQI for evaluating the parents’ sleep problems.

### 2.4. Data Analysis

We conducted our statistical analyses after confirming that the data were normally distributed using the Shapiro–Wilk test of continuous variables. The continuous variables were expressed as means (±standard deviations). The categorical variables were expressed as numbers (%). The participants were divided into two groups based on whether (DFT index ≥ 1) or not (DFT index = 0) they had any experience with caries. We compared the participants’ clinical characteristics based on their caries experience using an independent t-test or the chi-squared test. To investigate the correlation between sleeping status and caries in children, a univariate linear regression analysis was used. The correlations between the parents’ PSQI or the children’s sleeping hours and the numbers of caries in the children were assessed using Pearson’s correlation coefficients. Multiple regression analyses were performed for the numbers of caries in the children as the dependent variable and the parents’ PSQI, children’s sleeping hours, and other clinical characteristics as the independent variables. A multiple regression analysis with the stepwise backwards (Wald) method using factors with *p*-values < 0.10 in the univariate analysis was performed in order to calculate the impact of sleeping status on caries in children. To analyse the factors affecting the children’s caries experience, logistic regression analysis was used in order to examine the associations between the children’s caries experience and each variable, with the incidence of caries experience as the dependent variable and the clinical characteristics as the independent variables. The final logistic regression model was developed by backwards stepwise selection from all of the variables that were significantly associated with caries experience in the bivariate analyses (*p* < 0.05). A *p*-value < 0.05 indicated statistical significance. The statistical analyses were performed with JMP14.0 J software (SAS Institute Japan, Tokyo, Japan).

## 3. Results

Of the 537 parents and their children, 205 were excluded from the study. Among these 205 excluded children, 175 parents (32.6%) did not agree to participate and answer the questionnaire in this study and 30 were missing their dental screening data. Ultimately, 332 parents and their children were included in this study. In our cohort, 42 children (12.7%) had experience with caries. The baseline characteristics, according to whether or not the children had experienced caries, are shown in Table 1. Children without caries experience slept longer than those with caries experience (10.0 ± 1.0 h vs. 9.5 ± 0.9 h; *p* = 0.002). The prevalence of skipping breakfast and the amount of screen time were higher in children with caries experience than in those without, while the prevalence of extracurricular activities was lower in children with caries experience than in those without (*p* < 0.05 for all). Interestingly, TV watching time, screen time, and game-playing time were significantly longer in children with caries experience compared with those without caries experience (*p* < 0.05 for all). Conversely, there were no significant differences in the children’s age, sex, or BMI between the two groups. Regarding the parents’ characteristics, the parents of the children with caries experience were more likely to smoke, be unmarried, or have a lower education status when compared with the parents of the children without caries experience (*p* < 0.05 for all). The mean PSQI of the group with caries experience was 6.0 ± 2.8, which was significantly higher than that of the group without caries experience (4.9 ± 2.7; *p* = 0.010). HL was better in the group without caries experience than in those with caries experience (53.5 ± 7.2 vs. 56.0 ± 7.8; *p* = 0.040). However, both groups were above the 50 points suggested as the cut-off for HL.

**Figure 1:**
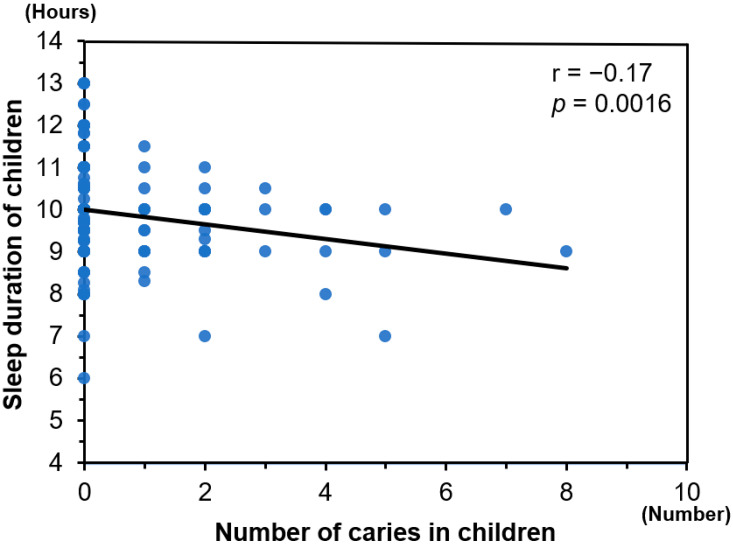
The relationship between sleep duration of children and number of caries in children.

**Figure 2:**
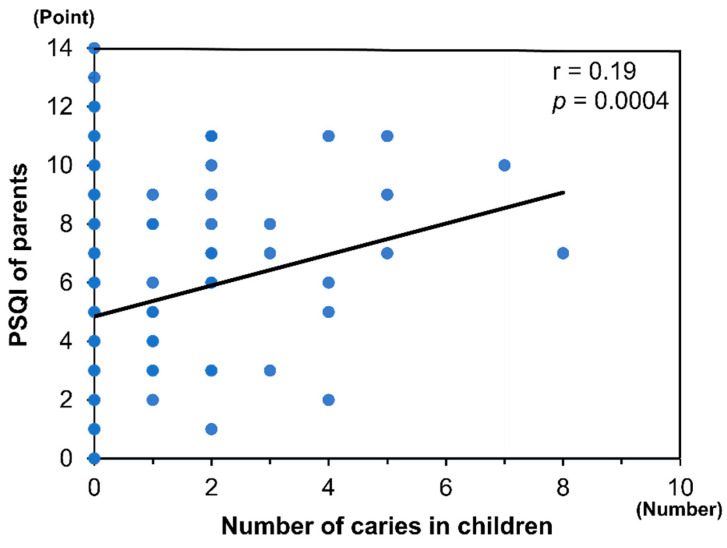
The relationship between PSQI of parents and number of caries in children.

There was a significant negative correlation between the sleep durations that were experienced by the children and the numbers of caries that they experienced (r = −0.17, *p* = 0.0016; Figure 1). In addition, the parents’ PSQI scores were significantly positively correlated with the numbers of caries in the children (r = 0.19, *p* = 0.0004; Figure 2). In the multiple linear regression analysis, both the sleep durations that were experienced by the children and the PSQI of their parents were independently associated with the numbers of caries in the children, after adjustment for confounding factors (*p* < 0.05 for all; Table S1).

Furthermore, the children’s screen time, parental smoking status, and parental marital status remained independent factors that were found to be associated with the numbers of caries in the children (*p* < 0.05 for all). Table 2 shows the results of the univariate and multivariate logistic regression models that were used for predicting the development of dental caries in the children. In the multivariate analysis, the sleep durations that were experienced by the children (adjusted odds ratio [OR] = 0.54), their screen time (adjusted OR = 1.35), and parental smoking status (adjusted OR = 3.91) were independently associated with the development of dental caries in the children. However, the parents’ PSQI did not reach statistical significance for predicting dental caries experience (adjusted OR = 1.06; *p* = 0.344).

## 4. Discussion

### 4.1. Main Findings of the Study

To the best of our knowledge, this is the first study to investigate the relationship between early childhood caries and the sleep statuses of the children and their parents. In our cohort, 12.7% of the children had experienced dental caries. After multivariate analysis, the sleep durations that were experienced by the children were identified as an independent risk factor for dental caries experience. Sleep problems experienced by their parents also tended to be associated with dental caries experience in children.

In this study, the sleep durations of the children and the sleep problems of their parents were associated with the dental caries experience of the children. Several mechanisms may play a role in these relationships. First, irregular and unhealthy lifestyles of parents such as skipping breakfast, excessive eating, eating too fast, excessive alcohol consumption, excessive number of hours spent watching TV, smoking, not exercising, eating unhealthy foods, and not maintaining a healthy weight may be associated with dental caries experience in children. Suma Sogi et al. [17] reported that a child’s sleep status is strongly associated with their parents’ behaviour. Parental behaviour or parents’ HL could be related to short sleep durations in children, leading to dental caries experience.

### 4.2. Findings of Previous Studies

Three previous studies have suggested that unhealthy lifestyle habits of parents, such as intake of sugary foods or drinks before going to bed or lack of oral hygiene practices, are likely to increase the likelihood of dental caries in children [17,18,19]. Low parental oral HL has been shown to play a significant role in preschool children’s experiences with caries [10]. Furthermore, Shantinath SD et al. demonstrated that nightly feeding is a significant risk factor for early childhood caries [20]. In our results, the prevalence of having a daily breakfast was lower in the group with caries experience. Furthermore, parents’ HL was lower in the group with caries experience, suggesting that a lack of HL could lead to early childhood caries. Furthermore, sleeping statuses or circadian rhythms are affected by daily physical and social activity [21]. Social connections and regular exercise habits may be influenced in order to maintain and improve circadian and sleep rhythms. As such, a lack of parents’ social capital could negatively impact their children’s health. Moreover, the deterioration of oral conditions due to a lack of sleep causes dental caries. A previous study has shown that sleep deprivation induces a decrease in salivary flow and an increase in the prevalence of *S. mutans*, a caries-causing bacteria [22]. Moreover, a shorter sleep duration increases the levels of pro-inflammatory cytokines [23] and decreases saliva production [22], resulting in an increased risk of dental caries in children. Furthermore, the WHO recommends that 10–13 h of sleep are needed for good quality sleep, including a nap, with regular sleep and wake-up times for children aged < 5 years. Our sample had a shorter sleeping duration compared with that which is recommended by the WHO [24]. It is possible that the children with caries might be suffering from sleep disturbances due to pain and discomfort [25].

A national survey of dental diseases in Japan demonstrated that the prevalence of dental caries in children was 8.6%, 36%, and 39% for 3, 4, and 5 year-olds, respectively [26]. The prevalence of dental caries in this study was 12.7%, which is relatively low compared with the national survey results. Previously, Julihn et al. [27] reported that later-born children are at a greater risk of developing caries compared with first-born children. The parents devote more attention to the first child, which leads to improved oral care for that child. Further, the more children in a family, the more family resources must be shared, which may decrease children’s performance [28]. This allows for the possibility that the incidence of early childhood caries was lower in our study than in other cohort studies because our study recruited only the oldest child in each family.

Besides sleeping duration, screen time and parental smoking status were identified as factors that are related to dental caries in the children in this study. A previous cohort study reported an independent relationship between the amount of time that was spent playing video games and the frequency of tooth brushing or dental caries in children aged 4–18 [29]. Another study revealed that excessive screen time was an independent predictor of dental caries in children aged 6–12 years [11]. This study supports the previous findings and implies that establishing a healthy lifestyle, including adequate sleep duration or avoiding excessive TV watching, gaming, and screen time, is necessary to prevent dental caries in children. Extended screen time can contribute to staying up late and screen time before bedtime may diminish children’s sleep quality. Subsequently, Gokhale and Nuvvula previously reported that children of lower socioeconomic status and children whose parents both have jobs are at a higher risk for dental caries [30]. Our results did not show a similar trend which relates parental smoking status and dental caries in children; however, a previous study had demonstrated that the dental caries prevalence in 3 year-olds was associated with parental smoking status [31]. However, these relationships may change depending on maternal or paternal smoking status. Interestingly, current adult smokers in Japan are more likely to be highly educated. There remain unanswered questions concerning the impact of parental smoking status on dental caries in children.

### 4.3. What This Study Adds

This study demonstrated, for the first time, a relationship between children’s dental caries and the sleeping statuses of the children and their parents. The sleep durations that were experienced by the children were independently associated with early childhood caries. Furthermore, the children’s dental caries were associated with parental sleep problems. This study suggests the need for guidance and a change in the lifestyle habits of parents in order to prevent dental caries in their children. Further studies should examine the effects of lifestyle modifications on oral health in children.

### 4.4. Limitations

This study had some limitations. First, it was a cross-sectional investigation and, therefore, the potential causal inference was limited. Second, because this was a questionnaire-based study, parents may have underreported certain items, which is the main drawback of questionnaire-based studies [32,33,34,35], and as such a recall or response biases may have occurred. Objective analysis, such as polysomnography, may be needed in order to evaluate sleep status and quality. Future large-scale trials are needed in order to investigate the comprehensive risk factors of dental caries and investigate the cut-off values and target values for preventing dental caries in children. Moreover, due to the small sample size, we could not analyse the relationship between the children’s age and their dental caries or the difference in background factors according to the numbers of caries in the children.

## 5. Conclusions

The findings of this study highlight the impacts of unfavourable lifestyles and behaviours on children’s oral health problems. The sleep durations that were experienced by the children were independently associated with early childhood caries. Furthermore, the occurrence of dental caries in children was associated with parental sleep problems. We believe that our study makes a significant contribution to the literature because it is the first to specifically examine the relationship between dental caries in children and the sleep statuses of those children and their parents. Further, we believe that this paper will be of interest as it highlights the importance of a healthy lifestyle for both parents and their children and we hope that it will encourage further studies into the specifics of the risk factors that we uncovered.

## Figures and Tables

**Table 1:** Variables of the child and parent and caries experience of the child.

	Total (*n* = 332)	with Caries Experience (*n* = 42)	without Caries Experience (*n* = 290)	*p* Value
**Child**				
Age, months	64.4 ± 10.1	66.9 ± 9.1	64.1 ± 10.2	0.089
Sex, female, *n* (%)	154 (46.4)	15 (35.7)	139 (47.9)	0.896
BMI, kg/m^2^	15.6 ± 1.5	15.9 ± 1.7	15.6 ± 1.5	0.158
Birth weight, g	3015.3 ± 435.5	3021.6 ± 370.8	3014.4 ± 444.6	0.920
Number of siblings, *n*	2.3 ± 0.8	2.5 ± 1.2	2.2 ± 0.8	0.094
Breakfast, every day, *n* (%)	311 (93.7)	36 (85.7)	275 (94.8)	0.023
Extracurricular activities, yes, *n* (%)	145 (43.7)	12 (28.6)	133 (45.9)	0.003
Sleep duration, h	9.9 ± 1.0	9.5 ± 0.9	10.0 ± 1.0	0.002
TV time, h	2.1 ± 1.2	2.6 ± 1.4	2.0 ± 1.2	0.003
Game time, h	0.5 ± 0.7	0.8 ± 0.9	0.5 ± 0.7	0.027
Screen time, h	2.6 ± 1.5	3.3 ± 1.9	2.5 ± 1.4	0.000
**Parent**				
Age, year	36.1 ± 5.3	35.4 ± 6.5	36.2 ± 5.1	0.387
Sex, female	311 (93.7)	41 (97.6)	270 (93.1)	0.261
BMI, kg/m^2^	21.5 ± 3.3	21.7 ± 4.4	21.4 ± 3.1	0.609
Smoking, yes, *n* (%)	44 (13.2)	13 (30.7)	31 (10.7)	0.003
Drinking, every day, *n* (%)	37 (11.1)	4 (9.5)	33 (11.4)	0.721
Marital status, married, *n* (%)	313 (94.2)	38 (85.7)	277 (95.0)	0.011
Working, yes, *n* (%)	233 (70.2)	30 (71.4)	203 (70.0)	0.850
Education, years	13.4 ± 1.6	12.7 ± 1.7	13.5 ± 1.6	0.003
Household income, >6 million, *n* (%)	119 (36.2)	11 (26.2)	108 (37.6)	0.1495
PSQI, points	5.0 ± 2.7	6.0 ± 2.8	4.9 ± 2.7	0.010
Health literacy, points	54.8 ± 7.3	53.5 ± 7.2	56.0 ± 7.8	0.040

Data are expressed as means ± standard deviations or numbers (percentages). BMI: body mass index; PSQI: Pittsburgh Sleep Quality Index.

**Table 2:** Univariate and multivariate analyses of risk factors associated with the development of dental caries in children.

Variables	Univariate Model		Multivariate Model	
	OR (95% CI)	*p* Value	OR (95% CI)	*p* Value
**Child**				
Sleep duration	0.58 (0.41–0.82)	0.0018	0.54 (0.36–0.80)	0.0012
Screen time	1.40 (1.15–1.70)	0.0010	1.35 (1.09–1.66)	0.0051
Age	1.03 (1.00–1.06)	0.0910		
Sex, female	0.60 (0.31–1.18)	0.1407		
Breakfast, not every day	3.06 (1.11–8.38)	0.0436		
Extracurricular activities	0.47 (0.23–0.96)	0.0314		
**Parent**				
Age	0.97 (0.92–1.03)	0.0274		
Sex, female	0.33 (0.04–2.52)	0.2847		
PSQI	1.16 (1.03–1.30)	0.014	1.06 (0.94–1.21)	0.344
Smoking, no	3.75 (1.76–7.95)	0.0011	3.91 (1.72–8.89)	0.0017
Marital status, no	3.55 (1.27–9.92)	0.0157		
Health literacy	1.05 (1.00–1.11)	0.0335		
Education	0.75 (0.62–0.91)	0.0039		

OR: odds ratio; CI: confidence interval; PSQI: Pittsburgh Sleep Quality Index.

## Data Availability

The data underlying this article will be shared on reasonable request to the corresponding author.

## Supplementary Materials

The following are available online at https://www.mdpi.com/article/10.3390/ejihpe12010001/s1, Table S1: Multiple regression analysis for predicting the number of caries in children; Table S2: STROBE Statement.
