# Supplementary material for: Association between Insufficient Sleep and Dental Caries among Preschoolers in Japan: A Cross-Sectional Multicentre Study
Source: Eur J Investig Health Psychol Educ. 2021 Dec 30;12(1):1–10. doi: 10.3390/ejihpe12010001 (PMC8774937; doi:10.3390/ejihpe12010001)
Supplement: Supplementary file 1 [file ejihpe-12-00001-s001.zip › ejihpe-1494231-supplementary.pdf]

Table S1. Multiple regression analysis for predicting the number of caries in children

	Number of caries		
	$R^2 = 0.14$; Adjusted $R^2 = 0.13$<br>$p < 0.0001$; F = 4.03		
	Standardised β	95% CI	*p* value
Sleep duration of children	-0.14	-0.25 to -0.04	0.0049
Screen time of children	0.15	0.04 to 0.18	0.0030
Smoking status of parents	-0.17	-0.40 to -0.10	0.0015
Marital status	-0.16	-0.59 to -0.14	0.0018
PSQI of parents	0.11	0.01 to 0.08	0.0455

CI: confidence interval; PSQI: Pittsburgh Sleep Quality Index

Table S2. STROBE Statement—Checklist of items that should be included in reports of ***cross-sectional studies***

	Item No	Recommendation
**Title and abstract**	1	(*a*) Indicate the study's design with a commonly used term in the title or the abstract<br>Page 1, Lines 2-3
		(*b*) Provide in the abstract an informative and balanced summary of what was done and what was found<br>Page 1, Lines 13-26
**Introduction**		
Background/rationale	2	Explain the scientific background and rationale for the investigation being reported<br>Page 1, Line 30- Page 2, Line 48
Objectives	3	State specific objectives, including any prespecified hypotheses<br>Page 2, Lines 49-55
**Methods**		
Study design	4	Present key elements of study design early in the paper<br>Page 2, Lines 57-59
Setting	5	Describe the setting, locations, and relevant dates, including periods of recruitment, exposure, follow-up, and data collection<br>Page 2, Lines 59-66
Participants	6	(*a*) Give the eligibility criteria and the sources and methods of selection of participants<br>Page 2, Lines 57-60
Variables	7	Clearly define all outcomes, exposures, predictors, potential confounders, and effect modifiers. Give diagnostic criteria, if applicable<br>Page 2, Line 71- P3 Line 103
Data sources/ measurement	8*	For each variable of interest, give sources of data and details of methods of assessment (measurement). Describe comparability of assessment methods if there is more than one group<br>Page 2, Line 71- Page 3, Line 103

Bias	9	Describe any efforts to address potential sources of bias<br>Page 2, Lines 59-63
Study size	10	Explain how the study size was arrived at<br>Page 2, Lines 59-62
Quantitative variables	11	Explain how quantitative variables were handled in the analyses. If applicable, describe which groupings were chosen and why<br>Page 3, Lines 105-109
Statistical methods	12	(*a*) Describe all statistical methods, including those used to control for confounding<br>Page 3, Lines 105-126
		(*b*) Describe any methods used to examine subgroups and interactions<br>Page 3, Lines 119-126
		(*c*) Explain how missing data were addressed<br>Page 3, Lines 128-130
**Results**		
Participants	13*	(a) Report numbers of individuals at each stage of study—eg numbers potentially eligible, examined for eligibility, confirmed eligible, included in the study, completing follow-up, and analysed<br>Page 3, Lines 128-130
		(b) Give reasons for non-participation at each stage<br>P3.Line 128-130
		(c) Consider use of a flow diagram<br>Page 3, Lines 128-131
Descriptive data	14*	(a) Give characteristics of study participants (eg demographic, clinical, social) and information on exposures and potential confounders<br>Page 3, Line 128- Page 4, Line 147
		(b) Indicate number of participants with missing data for each variable of interest<br>Page 3, Lines 128-130
Outcome data	15*	Report numbers of outcome events or summary measures<br>Page 3, Lines 131-132
Main results	16	(*a*) Give unadjusted estimates and, if applicable, confounder-adjusted estimates and their precision (eg, 95% confidence interval). Make clear which confounders were adjusted for and why they were included<br>None
		(*b*) Report category boundaries when continuous variables were categorized<br>Page 3, Lines 108-109
		(*c*) If relevant, consider translating estimates of relative risk into absolute risk for a meaningful time period<br>None
Other analyses	17	Report other analyses done—eg analyses of subgroups and interactions, and sensitivity analyses<br>Page 5, Lines 165-169
**Discussion**		
Key results	18	Summarise key results with reference to study objectives<br>Page 6, Lines 174-189
Limitations	19	Discuss limitations of the study, taking into account sources of potential bias or imprecision. Discuss both direction and magnitude of any potential bias<br>Page 7, Line 249- Page 8, Line 258
Interpretation	20	Give a cautious overall interpretation of results considering objectives, limitations, multiplicity of analyses, results from similar studies, and other

		relevant evidence<br>Page 6, Line 190- Page 7, Line 239
Generalisability	21	Discuss the generalisability (external validity) of the study results<br>Page 7, Lines 241-247.
**Other information**		
Funding	22	Give the source of funding and the role of the funders for the present study and, if applicable, for the original study on which the present article is based<br>Page 8, Line 272

*Give information separately for exposed and unexposed groups.

Note: An Explanation and Elaboration article discusses each checklist item and gives methodological background and published examples of transparent reporting. The STROBE checklist is best used in conjunction with this article (freely available on the Web sites of PLoS Medicine at http://www.plosmedicine.org/, Annals of Internal Medicine at http://www.annals.org/, and Epidemiology at http://www.epidem.com/). Information on the STROBE Initiative is available at www.strobe-statement.org.
